# First-Principles Study on Structural, Mechanical, Anisotropic, Electronic and Thermal Properties of III-Phosphides: *X*P (*X* = Al, Ga, or In) in the *P*6_4_22 Phase

**DOI:** 10.3390/ma13030686

**Published:** 2020-02-04

**Authors:** Junjie Miao, Changchun Chai, Wei Zhang, Yanxing Song, Yintang Yang

**Affiliations:** Key Laboratory of Ministry of Education for Wide Band-Gap Semiconductor Materials and Devices, School of Microelectronics, Xidian University, Xi’an 710071, China; miao_junjie@163.com (J.M.); ccchai@mail.xidian.edu.cn (C.C.); syx739686768@163.com (Y.S.); ytyang@xidian.edu.cn (Y.Y.)

**Keywords:** III-phosphide, *P*6_4_22 phase, density functional theory, mechanical properties, band-gap properties, thermal properties

## Abstract

The structural, mechanical, electronic, and thermal properties, as well as the stability and elastic anisotropy, of *X*P (*X* = Al, Ga, or In) in the *P*6_4_22 phase were studied via density functional theory (DFT) in this work. *P*6_4_22-*X*P (*X* = Al, Ga, or In) are dynamically and thermodynamically stable via phonon spectra and enthalpy. At 0 GPa, *P*6_4_22-*X*P (*X* = Al, Ga, or In) are more rigid than *F*$\bar{4}3$*m*-*X*P (*X* = Al, Ga, or In), of which *P*6_4_22-*X*P (*X* = Al or Ga) are brittle and *P*6_4_22-InP is ductile. In the same plane (except for (001)-plane), *P*6_4_22-AlP and *P*6_4_22-InP exhibit the smallest and the largest anisotropy, respectively, and *P*6_4_22-*X*P (*X* = Al, Ga, or In) is isotropic in the (001)-plane. In addition, Al, Ga, In, and P bonds bring different electrical properties: *P*6_4_22-InP exhibits a direct band gap (0.42 eV) with potential application for an infrared detector, whereas *P*6_4_22-*X*P (*X* = Al or Ga) exhibit indirect band gap (1.55 eV and 0.86 eV). At high temperature (approaching the melting point), the theoretical minimum thermal conductivities of *P*6_4_22-*X*P (*X* = Al, Ga, or In) are AlP (1.338 W∙m^−1^∙K^−1^) > GaP (1.058 W∙m^−1^∙K^−1^) > InP (0.669 W∙m^−1^∙K^−1^), and are larger than those of *F*$\bar{4}3$*m*-*X*P (*X* = Al, Ga, or In). Thus, *P*6_4_22-*X*P (*X* = Al, Ga, or In) have high potential application at high temperature.

## 1. Introduction

GaP and InP, which are typical second-generation compound semiconductor materials, are primarily used to produce high-speed, high-frequency, high-power, and light-emitting electronic devices. These materials are also excellent materials for producing high-performance microwave and millimeter-wave devices and light-emitting devices. With the rise of the information highway and the Internet, these materials have also been widely used in the fields of satellite and mobile communications, solar power technology and GPS navigation [1]. AlP is an important material that is mainly used in light emitting diodes and infrared photo detectors [2,3] in industrial application. AlP, GaP, and InP have been given wide attention due to high thermal conductivities and wide energy band gap [4].

Computational chemistry is the subject of applying computer technology based on basic physicochemical theories (quantum chemistry, statistical thermodynamics, and classical mechanics) and a large number of numerical methods to study and predict the regularity of the relationship between the structure and properties of chemicals. The present direction of material research and development should combine computational chemistry with material design, apply the basic principle of computational chemistry, and carry out material design and simulation on molecule and atom level to provide reliable theoretical guidance. The first-principle electronic structure method is widely used in materials science, including Hartree–Fork equation, DFT and so on.

Increasingly many researchers have focused on the polymorphism of *X*P (*X* = Al, Ga, or In) compound semiconductors. Xu et al. [5] studied the polymorphs, mechanical, and thermodynamic properties of Al*X* (*X* = N, P, or As) compound in the wurtzite, zinc-blende, and NiAs structures via first-principle calculations, and summarized the relationships between the temperature and the thermodynamic properties of Al*X* (*X* = N, P, or As) compounds based on a quasi-harmonic approximation. The results demonstrated that in the same structure, the hardness and Debye temperature decrease: AlN > AlP > AlAs. Based on the advanced method of crystal structure prediction, three new metastable structures of AlAs were investigated by Liu et al. [6], namely, *h*P6-AlAs, *o*C12-AlAs, and *c*I24-AlAs, of which the space groups are *P*6_4_22, *C*222, and *I*$\bar{4}3$*d*, respectively. The mechanical and dynamic stabilities of these structures were evaluated by calculating the elastic constant and the phonon spectrum. According to first-principle calculations, the hardness of *o*C12- and *h*P6-AlAs are larger than that of *c*I24-AlAs under the same pressure. Under ambient pressure, *o*C12-, *h*P6-AlAs, and *c*I24-AlAs exhibit semiconductor properties and the first two show direct band gap properties (0.468 eV and 1.356 eV), whereas the last exhibits indirect band gap property (1.761 eV).

By utilizing a crystal structure prediction software (CALYPSO), Yang Ruike [7] proposed four possible phases of AlP (*Pmn*2_1_-, *Pbam*-, *Pbca*-, and *bct*-AlP) and studied their structures, elastic constants, thermodynamics, and electrical properties based on first-principles. It was found that these four new phases all have semiconductor properties; *Pmn*2_1_-AlP and *Pbam*-AlP show direct band gap properties with larger electronic advantages than wz-AlP and zb-AlP at ambient pressure; and *Pmn*2_1_-AlP, *Pbam*-AlP, *Pbca*-AlP, and *bct*-AlP are ductile. *Pmn*2_1_-AlP and *Pbam*-AlP are direct band gap semiconductors (3.22 eV and 3.27 eV), whereas *Pbca*-AlP and *bct*-AlP are indirect band gap semiconductors (3.47 eV and 3.04 eV). Based on density functional theory (DFT), A. Baida et al. [8] studied the structural, optical, and electronic properties of indium phosphide (InP) via the augmented plane wave (FP-LAPW) method. The results demonstrated that the phase transitions from zinc-blende phase to Imm2, NiAs, PbO, and CsCl phases are possible at low pressure.

Arbouche et al. [9] used the full-potential linearized augmented plane-wave (FP-LAPW+lo) method to calculate the phase transitions of zinc-blende (zb), sc16, cmcm, NaCl, C_S_Cl, d-β-tin, Imm2, Immm, and NiAs of III-phosphide *X*P (*X* = Al, Ga, or In) under high pressure. The calculated physical parameters such as the lattice constants and bulk modulus demonstrated that zb-*X*P (*X* = Al, Ga, or In) are more stable than these phases and cmcm-*X*P (*X* = Al, Ga, or In) have the highest hardness, respectively. The results on pressure transitions demonstrated that GaP will transform from the zb phase to the NaCl phase at 22.19 GPa and into the Imm2 phase above 33.76 GPa. When the pressure changed, zb-AlP and zb-InP will transform into NaCl-AlP (at 11.78 GPa) and NaCl-InP (at 7.35 GPa), respectively, whereas C_S_Cl-AlP and C_S_Cl-InP transform into the NaCl-AlP (at 64.89 GPa) and NaCl-InP (at 71.79 GPa), respectively. 

The physical properties of *X*P (*X* = Al, Ga, or In) in the *P*6_4_22 phase have not been identified to date. Therefore, in this work, the initial geometries of *P*6_4_22-*X*P (*X* = Al, Ga, or In) are constructed by atomic substitution base on the structure of *h*P6-AlAs [6]. The structural, mechanical, thermal, and electronic properties and the stability of *P*6_4_22-*X*P (*X* = Al, Ga, or In) have been systematically studied via density functional theory. The results demonstrate that only *P*6_4_22-InP is a direct band gap semiconductor material with potential application in an infrared detector.

## 2. Calculation Methods

**Figure 1:**
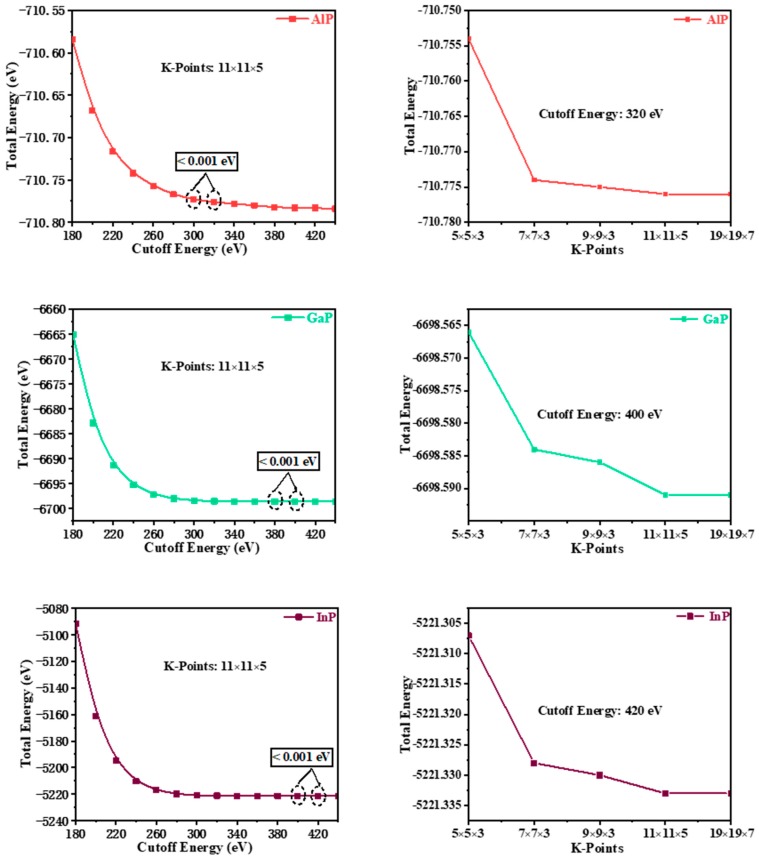
The convergence analysis of cut-off energy and the k-point grid allocation in the Brillouin zone.

The theoretical investigations on *P*6_4_22-*X*P (*X* = Al, Ga, or In) were conducted by utilizing density functional theory (DFT) [10,11], which is one of the most commonly used methods for calculating the properties of condensed matter physics based on the CASTEP code [12]. The generalized gradient approximation (GGA) [13] and the Perdew–Burke–Ernzerhof (PBE) [14] exchange-correlation functional were used for geometry optimization and property prediction of the materials. To improve computational precision, the convergence analysis of cut-off energy and the k-point grid allocation in the Brillouin zone are completed in turn by keeping the cut-off energy and the k-point constant, respectively. As is shown in Figure 1, the plane-wave cut-off energies were finally chosen to be 320, 400, and 420 eV with ultrasoft pseudopotentials for *P*6_4_22-AlP, *P*6_4_22-GaP, and *P*6_4_22-InP, respectively. The k-points in the first irreducible Brillouin zone were set to (11 × 11 × 5; 11 × 11 × 5; 11 × 11 × 5) [15] by using the Monkhorst–Pack scheme [16] for *P*6_4_22-AlP, *P*6_4_22-GaP, and *P*6_4_22-InP. By using the Broyden–Fletcher–Goldfarb–Shenno (BFGS) algorithm [17], structural parameter optimizations were conducted with the following thresholds for the convergent structures: a maximum stress of less than 0.02 GPa, a maximum residual force of less than 0.01 eV/Å, a maximum energy change of less than 5 × 10^−6^ eV per atom, and a maximum displacement of atoms for geometry optimization of less than 5 × 10^−4^ Å. The phonon spectra were calculated via linear response theory (density functional perturbation theory (DFPT)) [18]. The accurate electronic band-gap structures of *P*6_4_22-*X*P (*X* = Al, Ga, or In) were obtained via the Heyd–Scuseria–Ernzerhof (HSE06) [19,20] screened-exchange hybrid functional base on the previous geometry optimizations via GGA-PBE. The configurations of the valence electrons are 3*s*^2^3*p*^3^ for P, 3*s*^2^3*p*^1^ for Al, 3*d*^10^4*s*^2^4*p*^1^ for Ga, and 4*d*^10^5*s*^2^5*p*^1^ for In. 

## 3. Results and Discussion

### 3.1. Structural Properties

**Figure 2:**
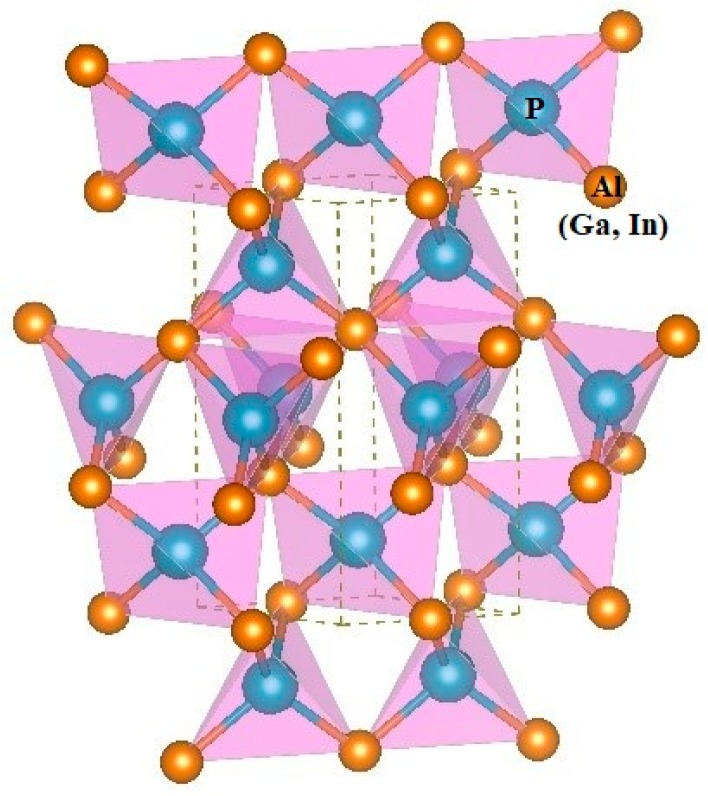
The 3D crystal structure of *P*6_4_22-*X*P (*X* = Al, Ga, or In).

The three-dimensional crystal structure of *P*6_4_22-*X*P (*X* = Al, Ga, or In) is illustrated in Figure 2. The 3D crystal structure of *P*6_4_22-*X*P (*X* = Al, Ga, or In) is composed of an sp^3^-bonded network. To evaluate the performance of the theoretical method that is used in this work, the related physical properties of *F*$\bar{4}3$*m*-*X*P (*X* = Al, Ga, or In) are also studied via the same method. The lattice parameters of *X*P (*X* = Al, Ga, or In) in the *P*6_4_22 phase and in the *F*$\bar{4}3$*m* phase are listed in Table 1 via GGA-PBE. The lattice parameters and the crystal density of *X*P (*X* = Al, Ga, or In) in the *F*$\bar{4}3$*m* phase (sphalerite phase) are very close to other experimental results, namely, the optimization and calculation method that is utilized in this work can provide theoretical support for the results [21,22,23]. In addition, the lattice structure of *P*6_4_22- and *F*$\bar{4}3$*m*-*X*P (*X* = Al, Ga, or In) are also optimized by using DFT-D2 (Grimme) on the basis of GGA-PBE to verify the effect of dispersion on the properties of the material. The results show that the errors between lattice constants *a*, *b*, and *c* of *F*$\bar{4}3$*m*-*X*P (*X* = Al, Ga, or In) and experimental values without (with) considering the dispersion action are 0.78% (0.46%), 0.99% (0.72%), 1.77% (0.26%), respectively, which proves our calculation method can provide theoretical support. For *P*6_4_22-*X*P (*X* = Al, Ga, or In), the lattice constants *a*, *b*, and *c* of *P*6_4_22-AlP change by ~1.53% (2.07% for *P*6_4_22-GaP, 3% for *P*6_4_22- InP), ~1.53% (2.07% for *P*6_4_22-GaP, 3% for *P*6_4_22-InP), and ~0.16% (0.2% for *P*6_4_22-GaP, 1.25% for *P*6_4_22-InP) with considering the dispersive action, indicating that *P*6_4_22-*X*P (*X* = Al, Ga, or In) are insensitive to the dispersive action. Considering the computational cost and accuracy, we adopt the optimized lattice parameters via GGA-PBE for subsequent studies of physical properties. The investigated *P*6_4_22-*X*P (*X* = Al, Ga, or In) has a hexagonal structure with the following equilibrium lattice parameters; *a* = *b* = 3.849 Å and *c* = 8.683 Å for AlP, *a* = *b* = 3.899 Å and *c* = 8.570 Å for GaP, and *a* = *b* = 4.190 Å and *c* = 9.416 Å for InP. For *P*6_4_22-*X*P (*X* = Al, Ga, or In), the P–Al bond length is 2.408 Å, the P–Ga bond length is 2.419 Å, and the P–In bond length is 2.618 Å. As shown in Table 1, in the same crystal structure, the volume per molecule for *P*6_4_22-*X*P (*X* = Al, Ga, or In) increases due to the long bond length and the large lattice constant. In the *P*6_4_22 phase, the densities of AIP (*ρ* = 2.591 g/cm^3^), GaP (*ρ* = 4.446 g/cm^3^) and InP (*ρ* = 5.073 g/cm^3^) are larger than the corresponding densities in the *F*$\bar{4}3$*m* phase because the corresponding volume per molecule in the *P*6_4_22 phase is smaller.

In Table 2, the equilibrium volume *V*_0_ and bulk modulus *B*_0_ of *P*6_4_22-*X*P (*X* = Al, Ga, or In) are calculated via GGA-PBE. The calculated total energy (*E*) per primitive cell for each compound as a function of different cell volumes (*V*) over a range of 0.9*V*_0_–1.1*V*_0_ is fitted by the Murnaghan equation of state [EOS] [21,22].

(1) $$E\left(V\right)=E_{0}+\frac{B_{0}V}{B^{\prime }\left(B^{\prime }-1\right)}\left[{\left(\frac{V_{0}}{V}\right)}^{B^{\prime }}+B^{\prime }\left(1-\frac{V_{0}}{V}\right)-1\right]$$

**Figure 3:**
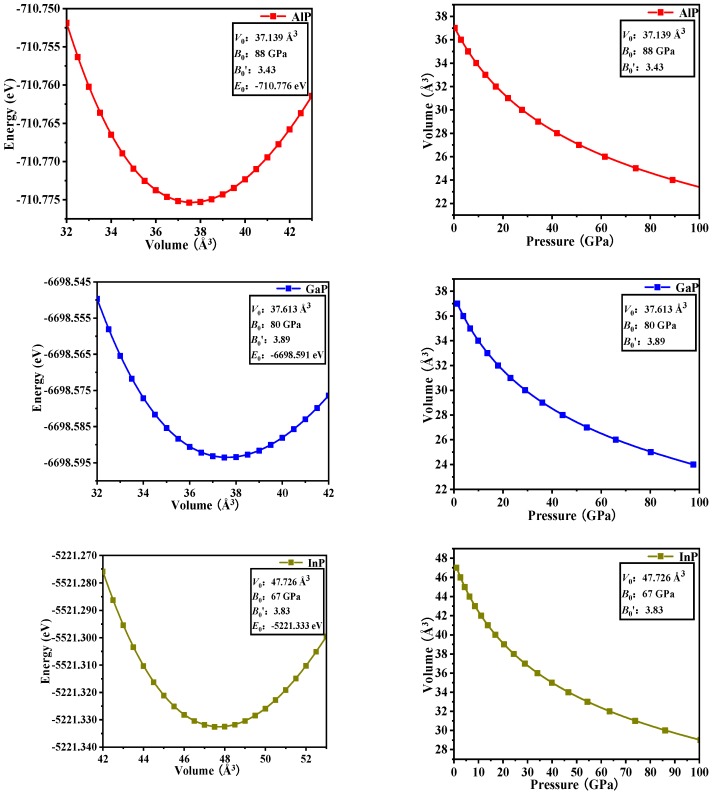
Computed total energy versus unit-cell volume (**left column**) and the variation of the volume versus pressure (**right column**) for *P*6_4_22-*X*P (*X* = Al, Ga, or In).

Where *B*_0_ and *B*′ are the bulk modulus and their first pressure derivatives at 0 GPa, *V*_0_ is the unit-cell volume at 0 GPa, and *E*(*V*) is the total energy under the different cell volume *V*. The fitted energy vs. volume (*E*-*V*) curves are shown in Figure 3. The equation between pressure and volume (*P*-*V* in Figure 3) is obtained through the derivation of *E*(*V*).

(2) $$P\left(V\right)=\frac{B_{0}}{B^{\prime }}\left[{\left(\frac{V_{0}}{V}\right)}^{B^{\prime }}-1\right]$$

In the fitting curve (*E*–*V*), there is a minimum energy near the volume *V*_0_, and this minimum energy (−710.776 eV for AlP, −6698.591 eV for GaP, and −5221.333 eV for InP) is in good agreement with the simulation data in Figure 1 (cut-off energy: 320, 400, and 420 eV, K -Points: 11 × 11 × 5, 11 × 11 × 5, 11 × 11 × 5 for *P*6_4_22-*X*P (*X* = Al, Ga, or In), respectively). It shows that *P*6_4_22-GaP are more stable than *P*6_4_22-*X*P (*X* = Al or In). Through the fitting *P*–*V* curve, InP*-P*6_4_22 has the largest volume compressibility: 38.15% (36.55% for AlP and 35.80% for GaP).

### 3.2. Stability and Mechanical Properties

**Figure 4:**
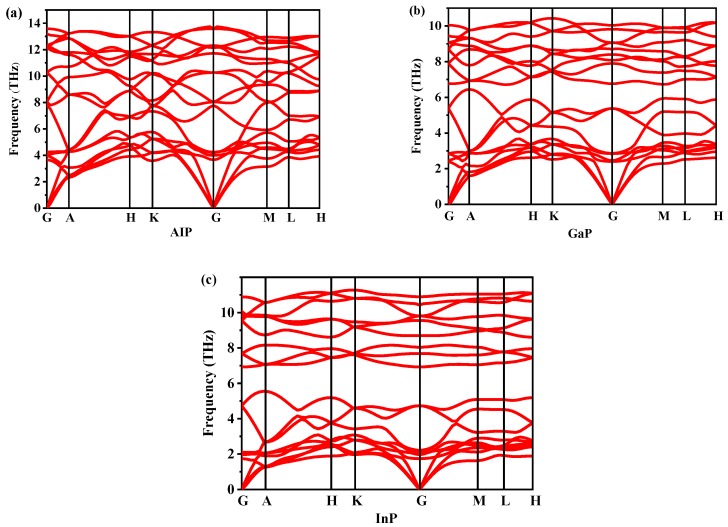
The phonon spectra of *P*6_4_22-*X*P (*X* = Al, Ga, or In): AlP (**a**), GaP (**b**), and InP (**c**).

Dynamic stability is an important property for verifying the existence of new materials. The dynamic stability of *P*6_4_22-*X*P (*X* = Al, Ga, or In) can be determined by studying the phonon spectra. The phonon spectra of *P*6_4_22-*X*P (*X* = Al, Ga, or In) are shown in Figure 4. By observation, the *P*6_4_22-*X*P (*X* = Al, Ga, or In) are dynamically stable because their phonon spectra have no imaginary frequencies in the Brillouin region. The highest vibrational frequencies of *P*6_4_22-*X*P (*X* = Al, Ga, or In) are 13.596 THz at point G, 10.412 THz at point K and 11.298 THz at point K, respectively. The elastic constants and elastic moduli of *P*6_4_22- and *F*$\bar{4}3$*m*-*X*P (*X* = Al, Ga, or In) are listed from 0 GPa to 35 GPa in Table 2. For *X*P (*X* = Al, Ga, or In) in the *F*$\bar{4}3$*m* phase, the calculated elastic constants are in good agreement with the reported experimental results, which proves the correctness of the theoretical calculation method. For a hexagonal system, the necessary and sufficient Born criteria for stability can be expressed as follows [26].

(3) $$C_{11}>0$$

(4) $$C_{11}>C_{12}$$

(5) $$(C_{11}+C_{12})C_{33}-2C_{13}^{2}>0$$

(6) $$C_{44}>0$$

In Table 2, all the elastic constants of *P*6_4_22-*X*P (*X* = Al, Ga, or In) at 0 GPa satisfy the above stability criteria, namely, *P*6_4_22-*X*P (*X* = Al, Ga, or In) are mechanically stable. The form ability and stability of the alloy can be characterized by the formation enthalpy and the cohesion energy [27]. To study the thermodynamic stability of *P*6_4_22-*X*P (*X* = Al, Ga, or In), its formation enthalpy (Δ*H*) and cohesive energy (*E*_coh_) are also further investigated, and the corresponding formulas [28,29] are described as follows,

(7) $$\Delta H=\left(E_{\mathrm{tot}}-N_{X}E_{\mathrm{solid}}^{X}-N_{P}E_{\mathrm{solid}}^{P}\right)/\left(N_{X}+N_{P}\right)$$

(8) $$E_{\mathrm{coh}}=\left(E_{\mathrm{tot}}-N_{X}E_{\mathrm{atom}}^{X}-N_{P}E_{\mathrm{atom}}^{P}\right)/\left(N_{X}+N_{P}\right)$$

where $E_{\mathrm{tot}}$ is the total energy of *P*6_4_22-*X*P (*X* = Al, Ga, or In) at the equilibrium lattice constant; $E_{\mathrm{solid}}^{X}$ and $E_{\mathrm{solid}}^{P}$ are the energies per atom of the pure constituents of *X* (*X* = Al, Ga, or In) and P, respectively, in the solid states; $E_{\mathrm{atom}}^{X}$ and $E_{\mathrm{atom}}^{P}$ are the energies from the free atoms of *X* (*X* = Al, Ga, or In) and P, respectively; and *N*_X_ and *N*_p_ refer to the numbers of *X* (*X* = Al, Ga, or In) and P atoms, respectively, in each conventional cell. The calculated formation enthalpies for *P*6_4_22-AlP, *P*6_4_22-GaP, and *P*6_4_22-InP are −1.72, −0.82, and −1.17 eV, respectively. All the values of formation enthalpies are negative; therefore, the bond energies of *P*6_4_22-*X*P (*X* = Al, Ga, or In) are very large and *P*6_4_22-*X*P (*X* = Al, Ga, or In) are easier to form, where *P*6_4_22-AlP > *P*6_4_22-InP > *P*6_4_22-GaP according to the stability of alloy formation. The cohesion energy is the energy that is needed for decomposing solid materials into isolated atoms. The smaller the value is, the higher the crystal structure stability. The results of *E*_coh_ for *X*P (*X* = Al, Ga, or In) in the *P*6_4_22 phase are −9.95, −8.21, and −8.74 eV, respectively. *P*6_4_22-AlP has the highest thermodynamic stability followed by *P*6_4_22-InP and, finally, *P*6_4_22-GaP, in a high-temperature environment.

The elastic moduli can be obtained based on the elastic constant. The bulk moduli *B* and the shear moduli *G* can be estimated via the Voigt–Reus–Hill approximation [30]. $B_{V}$, $B_{R}$, $G_{V}$ and $G_{R}$ can be expressed via the following equations [31], where the subscripts V and R are the Voight and Reuss schemes:

(9) $$B_{V}=(1/9)[C_{11}+C_{22}+C_{33}+2(C_{12}+C_{13}+C_{23})]$$

(10) $$B_{R}=\Delta {\left[C_{11}(C_{22}+C_{33}-2C_{23})+C_{22}(C_{33}-2C_{13})-2C_{33}C_{12}+C_{12}(2C_{23}-2C_{12})+C_{23}(2C_{13}-2C_{23})\right]}^{-1}$$

(11) $$G_{V}=(1/15)[C_{11}+C_{22}+C_{33}+3(C_{44}+C_{55}+C_{66})-(C_{12}+C_{13}+C_{23})]$$

(12) $$\begin{array}{ll} G_{R}= & 15\{4[C_{11}(C_{22}+C_{33}+C_{23})+C_{22}(C_{33}+C_{13})+C_{33}C_{12}-C_{12}(C_{23}+C_{12})-\\ & C_{13}(C_{12}+C_{13})-C_{23}(C_{13}+C_{23})]/\Delta +3[(1/C_{44})+(1/C_{55})+(1/C_{44})]{\}}^{-1} \end{array}$$

(13) $$\Delta =C_{13}(C_{12}C_{23}-C_{13}C_{22})+C_{23}(C_{12}C_{13}-C_{23}C_{11})+C_{33}(C_{11}C_{22}-C_{12}^{2})$$

(14) $$B=(1/2)(B_{V}+B_{R})$$

(15) $$G=(1/2)(G_{V}+G_{R})$$

Young’s modulus *E* and Poisson’s ratio *ʋ* are calculated from *B* and *G* as

(16) E=9BG/(3B+G)

(17) υ=(3B−2G)/[2(3B+G)]

According to Table 2, the elastic constants *C*_11_ (147 GPa, 152 GPa, 108 GPa), *C*_22_ = *C*_11_ (147 GPa, 152 GPa, 108 GPa), and *C*_33_ (174 GPa, 144 GPa, 117 GPa) for *P*6_4_22-*X*P (*X* = Al, Ga, or In) are larger than *C*_11_ = *C*_22_ = *C*_33_ (123 GPa, 134 GPa, 96 GPa) of *F*$\bar{4}3$*m*-*X*P (*X* = Al, Ga, or In); therefore, *P*6_4_22-*X*P (*X* = Al, Ga, or In) have stronger ability to resist elastic deformation along the X-, Y-, and Z- axes. The bulk moduli *B* and the shear moduli *G* of *P*6_4_22-*X*P (*X* = Al or In) are larger than those of *F*$\bar{4}3$*m*-*X*P (*X* = Al or In); thus, the anti-compression and anti-shearing strain abilities of *P*6_4_22-*X*P (*X* = Al or In) are stronger. Furthermore, the B/G ratios [32] of *P*6_4_22- and *F*$\bar{4}3$*m-X*P (*X* = Al, Ga, or In) at ambient pressure are also shown in Table 2. In the *P*6_4_22 phase, *X*P (*X* = Al or Ga) are brittle (*B/G* < 1.75) and InP are ductile (*B/G* > 1.75), and *F*$\bar{4}3$*m*-*X*P (*X* = Al, Ga, or In) are all brittle (*B/G* < 1.75). 

The calculated Young’s modulus *E* of *X*P (*X* = Al, Ga, or In) in the *P*6_4_22 phase at 0 GPa are 132, 140 and 94 GPa, respectively, which are larger than those (118, 131, and 88 GPa) in the *F*$\bar{4}3$*m* phase. Therefore, the stiffness of *P*6_4_22-*X*P (*X* = Al, Ga, or In) are higher, and they are more difficult to deform, especially GaP. There are no significant changes in the calculated values of Poisson’s ratio *ʋ* of *X*P (*X* = Al, Ga, or In) between the *P*6_4_22 phase and *F*$\bar{4}3$*m* phase at 0 GPa. The Poisson’s ratios *ʋ* of *P*6_4_22-AlP and *P*6_4_22-InP are 0.25 and 0.27, which are slightly larger than that of GaP (0.21) in the *P*6_4_22 phase. All Poisson’s ratios *ʋ* of *P*6_4_22-*X*P (*X* = Al, Ga, or In) are less than 1; thus, after the *P*6_4_22-*X*P (*X* = Al, Ga, or In) are subjected to uniform longitudinal stress, the transverse deformations are smaller than the longitudinal deformations before plastic deformation occurs, especially for GaP.

Pressure is a significative physical parameter that has a momentous impact on the Brillouin zone. Enthalpy is an important state parameter in thermodynamics for characterizing the energy of a material system. The lower its energy of matter or a system, the less likely it is to undergo spontaneous processes; therefore, the more stable it is [33].

**Figure 5:**
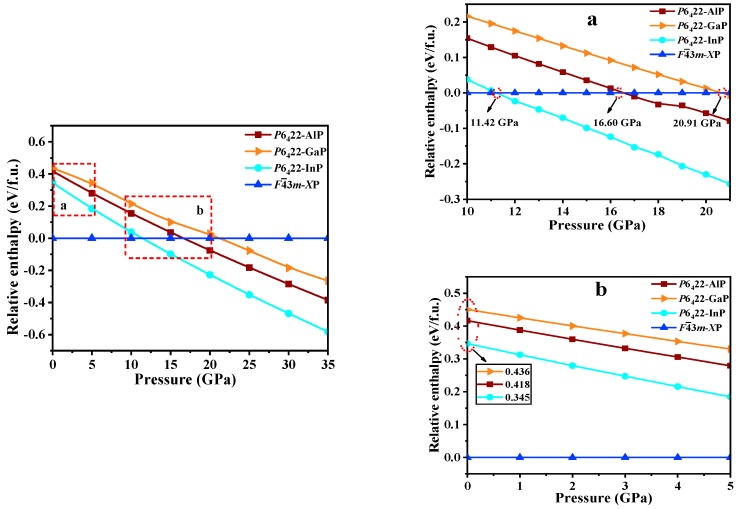
The relative formation enthalpies curves (relative to *F*$\bar{4}3$*m*-*X*P) as a function of pressure (0 to 35 GPa) for *P*6_4_22-*X*P (*X* = Al, Ga, or In); (**a**,**b**) are the zoomed in views of selected areas.

The relative formation enthalpy curves relative to *F*$\bar{4}3$*m*-*X*P (*X* = Al, Ga, or In) as functions of the pressure up to 35 GPa for *P*6_4_22-*X*P (*X* = Al, Ga, or In) are plotted in Figure 5. At ambient pressure, *F*$\bar{4}3$*m*-*X*P (*X* = Al, Ga, or In) are more favorable than any other *P*6_4_22-*X*P. Moreover, at 0 GPa, *P*6_4_22-AlP, *P*6_4_22-GaP, and *P*6_4_22-InP have larger enthalpy than *F*$\bar{4}3$*m*-*X*P (*X* = Al, Ga, or In) (0.418, 0.436, and 0.345 eV per formula (f.u.), respectively). As the pressure increases, *P*6_4_22-*X*P (*X* = Al, Ga, or In) become increasingly stable, and *P*6_4_22-AlP, *P*6_4_22-GaP, and *P*6_4_22-InP become more stable than *F*$\bar{4}3$*m*-AlP, *F*$\bar{4}3$*m*-GaP, and *F*$\bar{4}3$*m*-InP at the pressures that exceed 11.42, 16.60, and 20.91 GPa, respectively. In addition, *P*6_4_22-InP is the most stable, followed by *P*6_4_22-AlP and, finally, *P*6_4_22-GaP. According to the Table 2, the values of the elastic constant, Young’s modulus *E* (GPa), and Poisson’s ratio *ʋ* increase with the pressure.

### 3.3. Mechanical Anisotropic Properties

The universal anisotropic index *A*^U^ that present the elastic anisotropy of *P*6_4_22-*X*P (*X* = Al, Ga, or In) also calculated for further investigation in this work. The relevant calculation formulas are given in [37]. In Table 2, the *A*^U^ of *P*6_4_22-*X*P (*X* = Al, Ga, or In) shows an increasing tendency with increasing atomic order (AI < Ga < In) at ambient pressure. The variation tendencies of *A*^U^ for *X*P (*X* = Al, Ga, or In) in the *P*6_4_22 phase differ from those of Young’s modulus *E*. For example, *P*6_4_22-InP has the smallest Young’s modulus in the *P*6_4_22-*X*P (*X* = Al, Ga, or In) but has the largest universal anisotropic index *A*^U^.

**Figure 6:**
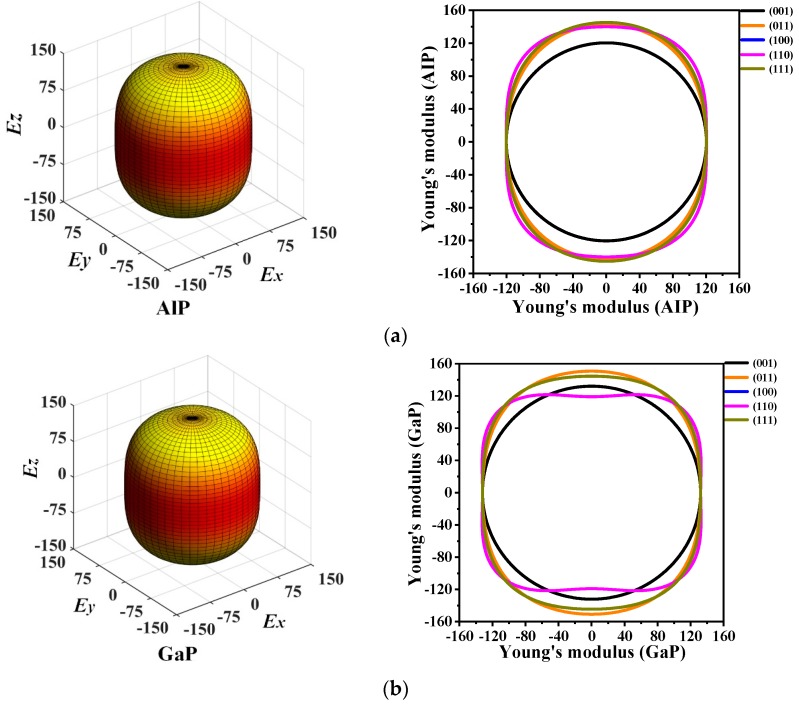

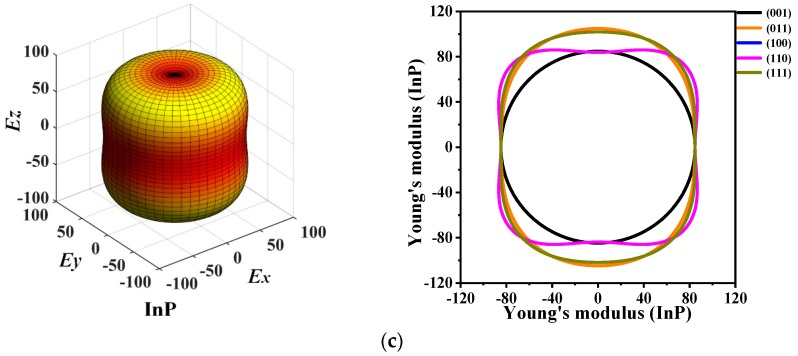
The 3D directional constructions and 2D representation of Young’s modulus *E* in the (001)-, (011)-, (100)-, (110)-, and (111)- plane for *P*6_4_22-AlP (**a**), P6_4_22-GaP (**b**) and P6_4_22-InP (**c**).

The 3D directional constructions and 2D representations of Young’s modulus *E* in the (001)-plane, (011)-plane, (100)-plane, (110)-plane, (010)-plane, and (111)-plane for *P*6_4_22-*X*P (*X* = Al, Ga, or In) are shown in Figure 6. Through observation, along with XY-, XZ-, and YZ-plane, *P*6_4_22-*X*P (*X* = Al, Ga, or In) exhibit strong anisotropy in various planes excluding XY-plane. Compared with the XY-plane, the three-dimensional surface structure in the XZ-plane deviates further from the shape of the sphere; therefore, the XZ- plane has stronger anisotropy than the XY-plane [38]. For *P*6_4_22-*X*P (*X* = Al, Ga, or In), the maximum and minimum values of Young’s modulus *E* are attained in the XZ- and YZ-planes, whereas only the minimum value is attained in the XY-plane because they are isotropic in the (001)-plane. In Figure 6, as Young’s modulus has the same properties in the (100)-, (010)-, and (110)-plane, Figure 6 shows only the two-dimensional curve in the (110)-plane.

The calculated maximum values *E*_max_, minimum values *E*_min_, and ratios *E*_max_/*E*_min_ of Young’s modulus *E* in each plane for *P*6_4_22-*X*P (*X* = Al, Ga, or In) are listed in Table 3. It is found that, in the (001)-plane, the minimum values of *E*_max_/*E*_min_ for *P*6_4_22-*X*P (*X* = Al, Ga, or In) are all 1.000; thus, *P*6_4_22-*X*P (*X* = Al, Ga, or In) are attained with the isotropy in the (001)-plane. The maximum ratio *E*_max_/*E*_min_ of Young’s modulus *E* is 1.206, with the largest anisotropy occurring in the (100)-, (110)-, and (010)-plane for *P*6_4_22-AlP. For *P*6_4_22-GaP, the maximum value of *E*_max_/*E*_min_ is 1.273, which is attained in the (100)-, (110)-, and (010)-plane with larger anisotropy. The ratios *E*_max_/*E*_min_ for *P*6_4_22-InP are all 1.251 in the (100)-, (110)-, and (010)-plane, which is larger than in the other planes. Therefore, the (100)-, (110)-, and (010)-plane of *P*6_4_22-InP exhibit higher anisotropy. In the (100)-, (110)-, and (010)-plane for *P*6_4_22-*X*P (*X* = Al, Ga, or In), the ratios *E*_max_/*E*_min_ of Young’s modulus are 1.206, and 1.251, respectively. In the (100)-, (110)-, and (010)-plane, *P*6_4_22-AlP exhibits the smallest anisotropy and *P*6_4_22-GaP exhibits the largest anisotropy. From the (011)-plane to the (111)-plane, *P*6_4_22-InP exhibits the largest anisotropy with *E*_max_/*E*_min_ = 1.237, and *P*6_4_22-GaP exhibits the smallest anisotropy with *E*_max_/*E*_min_ = 1.147.

### 3.4. Electrical and Thermal Properties

**Figure 7:**
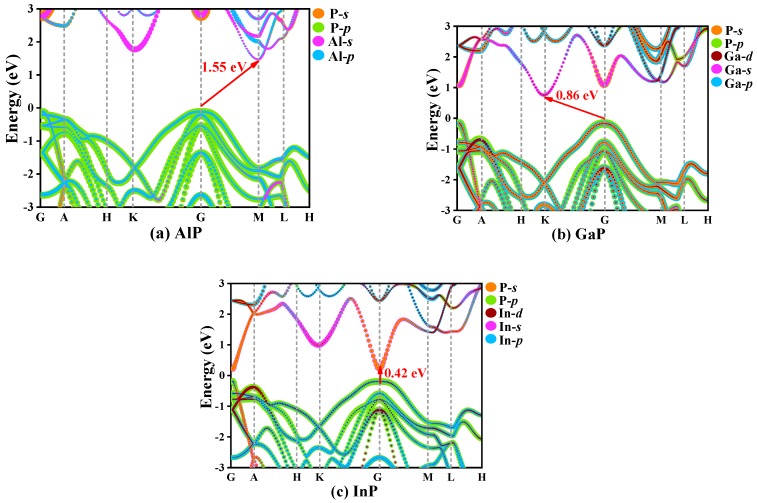
The electronic band structure for *P*6_4_22-*X*P (*X* = Al, Ga, or In), AlP (**a**), GaP (**b**), InP (**c**).

In solid-state physics, the electron band structure describes the energy that electrons are prohibited or allowed to carry, which is caused by quantum dynamic electron wave diffraction in periodic lattices [39]. The general characteristics of electron motion in crystals are qualitatively expounded by energy band theory. The orbital projection electronic band structures for *P*6_4_22-*X*P (*X* = Al, Ga, or In) are plotted in Figure 7. The coordinates of high-symmetry points in the Brillouin zone for *P*6_4_22-*X*P (*X* = Al, Ga, or In) are G (0.00, 0.00, 0.00), A (0.00, 0.00, 0.50), H (−0.33, 0.67, 0.50), K (0.33, 0.67, 0.00), G (−0.50, 0.50, 0.00), M (0.00, 0.50, 0.00), L (0.00, 0.50, 0.50), and H (−0.33, 0.67, 0.50). The band structures of *P*6_4_22-*X*P (*X* = Al, Ga, or In) are calculated via the HSE06 hybrid functional [40]. In the *P*6_4_22 phase, only InP is a direct band gap semiconductor, which has a band gap of 0.42 eV and the conduction band minimums and the valence band maximums are both located at point G (0.00, 0.00, 0.00). The band gap of *P*6_4_22-InP corresponds to a wavelength of 2958.04 nm, which is in the infrared region. *P*6_4_22-AlP and *P*6_4_22-GaP show indirect band gap properties with band gaps of 1.55 and 0.86 eV, respectively. The conduction band minimums and the valence band maximums of *P*6_4_22-AlP are located at point G (0.00, 0.00, 0.00) and point M (0.00, 0.50, 0.00), respectively, whereas the conduction band minimums and the valence band maximums of *P*6_4_22-GaP are located at point G (0.00, 0.00, 0.00) and point K (0.33, 0.67, 0.00), respectively.

**Figure 8:**
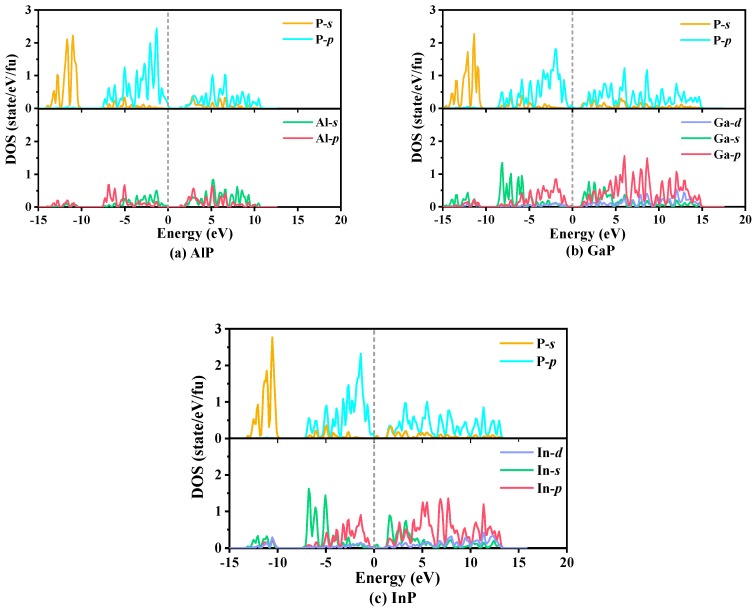
The partial densities of states of *P*6_4_22-*X*P (*X* = Al, Ga, or In): AlP (**a**), GaP (**b**), and InP (**c**).

The calculated partial atomic site projected densities of states (PDOS) of *P*6_4_22-*X*P (*X* = Al, Ga, or In), which are used to reflect elastic characteristics and the bonding properties and orbital distribution of electrons, are plotted in Figure 8. The main bonding peaks distribute in the range from −15 to 15 eV. Below 0 eV, the PDOS in the valence band consist of three parts: the first part ranges from −5 to −10 eV, where the −*s* orbital makes a larger contribution to electrical conductivity, and, in this part, the percentages of the −*p* orbital change minimally with increasing energy; the second part ranges from −10 to −5 eV, where the main contributions to conduct electricity are from the −*p* orbital for AlP, whereas the main contributions to conduct electricity are from the −*s* orbital for GaP and InP; and the last part consists of the −*p* orbital from −5 to 0 eV. Above 0 eV, the PDOS in the conduction band originate mainly consist of the −*p* orbital. From AlP to *X*P (*X* = Ga or In), due to the increase in the atomic volume, the contributions of the −*s* orbital increase substantially from the Al atom to the *X* (*X* = Ga or In) atoms in the range of −10 to −5 eV, and when the energy exceeds −5 eV, the contributions of the −*p* orbital increase substantially. In addition, in the vast majority of the energy range, the PDOS originate mainly from the −*p* orbital, namely, strong hybridization from the −*p* orbital of the P atom and the −*p* orbital of the *X* (*X* = Al, Ga, or In) atoms occurs. These PDOS peaks depend on the *X*–*p*/P–*p* (*X* = Al, Ga, or In) bonding orbital contribution. The results demonstrate that covalent bonds *X*–P (*X* = Al, Ga, or In) interactions occur.

Finally, we examine the theoretical minimum thermal conductivity under high temperature, representing the heat that is transferred through the phonon transmission in a temperature gradient, which depends not only on material thermal conductivity, but also on the temperature at which the material attains the lowest thermal conductivity, namely, the minimal thermal conductivity of the material. According to Clark, the main factors that affect it are the average relative atomic mass, the Young’s modulus, the density, the defects in the crystal, and the porosity. In addition, Cahill posits that the wave velocity of the acoustic wave is also closely related to the thermal conductivity of the material, and as the thermal conductivity decreases with the increase of the temperature under high-temperature conditions, its minimum value is of substantial significance to the application of the material under the high-temperature conditions. The theoretical minimum thermal conductivity is calculated via the Clark [41] model and the Cahill [42] model.

Clark model:

(18) $$\kappa_{\min}=0.87k_{B}M_{a}^{-2/3}E^{1/2}\rho^{1/6}$$

Cahill model:

(19) $$\kappa_{\min}=\frac{k_{B}}{2.48}p^{2/3}\left(v_{l}+2v_{t}\right)$$

In the Clark model, *E* and *ρ* represent the Young’s modulus and density of the crystal, respectively; *k*_B_ represents the Boltzmann constant; and *M*_a_ = [*M*/(n ∙ *N*_A_)] represents the average mass of the atoms in the lattice, where *M* is the molar mass of the molecule, n is the number of atoms in the molecule, and *N*_A_ represents Avogadro’s constant. In the Cahill model, *p* is the number of atoms per unit volume, and ν_l_ and ν_t_ [43] are the average acoustic longitudinal wave and acoustic shear wave, respectively, which can be calculated via the following formulas.

(20) $$v_{l}=\sqrt{(B+4G/3)/\rho }$$

(21) $$v_{t}=\sqrt{G/\rho }$$

The calculation results are presented in Table 4, in accordance with Formulas (18) and (19), and the theoretical minimum thermal conductivities of *P*6_4_22-AlP, *P*6_4_22-GaP, and *P*6_4_22-InP in the Clark model are 1.222 W∙m^−1^∙K^−1^, 0.972 W∙m^−1^∙K^−1^, and 0.610 W∙m^−1^∙K^−1^, respectively. In the Cahill model, the theoretical minimum thermal conductivities for *P*6_4_22-*X*P (*X* = Al, Ga, or In) are 1.338 W∙m^−1^∙K^−1^, 1.058 W∙m^−1^∙K^−1^ and 0.669 W∙m^−1^∙K^−1^, respectively. According to the calculated values, the theoretical minimum values of the thermal conductivity that are calculated by the Clark model are slightly less than those by the Cahill model. As the contributions of the atomic number density and the phonon spectrum are considered in the Cahill model, whereas the Clark model does not calculate the contribution of the optical phonons [44], the Clark model underestimates the theoretical minimum thermal conductivity and the Cahill model yields a value that is closer to the actual value. The maximum of the theoretical minimum thermal conductivity of *P*6_4_22-*X*P (*X* = Al, Ga, or In) corresponds to *P*6_4_22-AlP and the minimum to *P*6_4_22-InP, namely, according to the capacity of heat dissipation at high temperature (approaching the melting point), *P*6_4_22-AlP > *P*6_4_22-GaP > *P*6_4_22-InP. The theoretical minimum thermal conductivities of *F*$\bar{4}3$*m*-*X*P (*X* = Al, Ga, or In) at high temperature are lower than those of *P*6_4_22-*X*P (*X* = Al, Ga, or In); therefore, *P*6_4_22-*X*P (*X* = Al, Ga, or In) have stronger thermal conductivity than *F*$\bar{4}3$*m*-*X*P (*X* = Al, Ga, or In) at high temperature.

## 4. Conclusions

In this study, the related properties of *P*6_4_22-*X*P (*X* = Al, Ga, or In) are investigated via the density functional method, which include structural, mechanical, anisotropy, electrical, and thermal properties. *P*6_4_22-*X*P (*X* = Al, Ga, or In) are dynamically, mechanically, and thermodynamically stable, where *P*6_4_22-*X*P (*X* = Al or In) show stronger anti-compression and anti-shearing strain abilities than *F*$\bar{4}3$*m*-*X*P (*X* = Al or In). In the *P*6_4_22 phase, *X*P (*X* = Al or Ga) are brittle, and InP is ductile. The stiffness of *P*6_4_22-*X*P (*X* = Al, Ga, or In) are higher, and they are more difficult to deform than *F*$\bar{4}3$*m*-*X*P (*X* = Al, Ga, or In), especially GaP. As the pressure increases, *P*6_4_22-*X*P (*X* = Al, Ga, or In) become increasingly stable. *P*6_4_22-*X*P (*X* = Al, Ga, or In) have the largest anisotropy in the (100)-plane and show isotropy in the (001)-plane. *P*6_4_22-InP is a direct band gap semiconductor, which has a band gap of 0.42 eV and potential application as an infrared detector. *P*6_4_22-*X*P (*X* = Al or Ga) exhibit indirect band gap properties with band gaps of 1.55 and 0.86 eV, respectively. At high temperature, *P*6_4_22-*X*P (*X* = Al, Ga, or In) have stronger thermal conductivity than *F*$\bar{4}3$*m*-*X*P (*X* = Al, Ga, or In), where maximum and minimum thermal conductivities correspond *P*6_4_22-AlP and *P*6_4_22-InP, respectively. These properties provide a theoretical basis and new ideas for the application of *P*6_4_22-*X*P (*X* = Al, Ga, or In) in optoelectronic devices and thermoelectric materials.

## Figures and Tables

**Table 1:** The calculated (GGA-PBE and DFT-D2) lattice parameters and densities of *P*6_4_22- and *F*$\bar{4}3$*m*-*X*P (*X* = Al, Ga, or In).

	Space Group	Methods	*a* [Å]	*c* [Å]	*V* [Å^3^ molecule^−1^]	*ρ* [g cm^−3^]
AlP	*P*6_4_22	PBE	3.849	8.683	37.139	2.591
		DFT-D2	3.790	8.669	35.942	2.678
	*F* $\bar{4}3$ *m*	PBE	5.510		41.822	2.301
		DFT-D2	5.442		40.297	2.388
	*F* $\bar{4}3$ *m* ^[a]^	Exp.	5.467		40.773	2.360
GaP	*P*6_4_22	PBE	3.899	8.570	37.613	4.446
		DFT-D2	3.818	8.553	35.996	4.646
	*F* $\bar{4}3$ *m*	PBE	5.505		41.717	4.009
		DFT-D2	5.412		39.631	4.220
	*F* $\bar{4}3$ *m* ^[b]^	Exp.	5.451		40.488	4.130
InP	*P*6_4_22	PBE	4.190	9.416	47.726	5.073
		DFT-D2	4.064	9.298	44.330	5.461
	*F* $\bar{4}3$ *m*	PBE	5.973		53.263	4.545
		DFT-D2	5.854		51.162	4.876
	*F* $\bar{4}3$ *m* ^[c]^	Exp.	5.869		50.540	4.790

^[a]^ Ref. [23]. ^[b]^ Ref. [24]. ^[c]^ Ref. [25].

**Table 2:** The calculated elastic constants (*C*_11_, *C*_12_, *C*_13_, *C*_33_, *C*_44_, *C*_66_), bulk moduli *B*, shear moduli *G*, Young’s modulus *E* (GPa), Poisson’s ratios *ʋ* and universal anisotropic index *A*^U^ for *P*6_4_22-*X*P (*X* = Al, Ga, or In) when pressure P (GPa) increases from 0 to 35 GPa via the method of GGA-PBE.

Space Group	Methods	P	*C*_11_	*C*_12_	*C*_13_	*C*_33_	*C*_44_	*C*_66_	*B*	*G*	*B/G*	*E*	*ʋ*	*A* ^U^
*P*6_4_22-AlP	PBE	0	147	51	58	174	60	48	88	53	1.68	132	0.25	0.064
		5	169	67	77	190	66	51	107	56	1.91	143	0.28	0.095
		10	190	83	96	224	64	53	127	58	2.19	151	0.30	0.060
		15	207	98	114	245	59	54	144	56	2.57	149	0.33	0.032
		20	226	113	132	267	57	56	161	57	2.82	153	0.34	0.029
		25	240	128	148	290	55	56	177	56	3.16	152	0.36	0.039
		30	257	143	166	313	50	57	194	55	3.53	151	0.37	0.064
		35	267	161	181	333	38	53	208	48	4.33	134	0.39	0.235
*F*$\bar{4}3$*m*-AlP	PBE	0	123	58			60		80	47	1.70	118	0.25	0.494
*F*$\bar{4}3$*m*-AlP ^[a]^	Exp.	0	129	56			52							
*P*6_4_22-GaP	PBE	0	152	37	49	144	67	57	80	58	1.38	140	0.21	0.087
		5	178	54	56	150	75	62	92	64	1.44	156	0.22	0.117
		10	213	82	116	228	82	65	140	66	2.12	171	0.30	0.234
		15	230	91	115	222	88	69	147	70	2.10	181	0.29	0.217
		20	250	108	111	207	92	71	151	73	2.07	189	0.29	0.211
		25	271	125	143	248	96	73	179	75	2.39	197	0.32	0.245
		30	291	140	167	273	102	76	200	76	2.63	202	0.33	0.319
		35	310	156	181	285	104	77	216	77	2.81	206	0.34	0.332
*F*$\bar{4}3$*m*-GaP	PBE	0	134	60			70		80	59	1.39	131	0.21	0.500
*F*$\bar{4}3$*m*-GaP ^[b]^	Exp.	0	141	62			70							
*P*6_4_22-InP	PBE	0	108	37	49	117	45	36	67	37	1.81	94	0.27	0.124
		5	130	53	64	135	48	38	84	40	2.10	104	0.29	0.120
		10	151	76	94	171	48	38	110	40	2.75	107	0.34	0.157
		15	168	93	108	187	55	37	126	42	3.00	113	0.35	0.257
		20	190	112	129	209	59	39	146	43	3.40	117	0.37	0.298
		25	211	127	148	230	53	42	165	44	3.75	121	0.38	0.164
		30	225	148	168	251	48	38	186	41	4.54	115	0.40	0.118
		35	245	161	188	273	56	42	201	44	4.57	123	0.40	0.233
*F*$\bar{4}3$*m*-InP	PBE	0	96	55			49		59	35	1.69	88	0.25	0.924
*F*$\bar{4}3$*m*-InP ^[c]^	Exp.	0	102	56			47							

^[a]^ Ref. [34]. ^[b]^ Ref. [35]. ^[c]^ Ref. [36].

**Table 3:** The calculated maximum values *E*_max_, minimum values *E*_min_ and ratios *E*_max_/*E*_min_ of *X*P (*X* = Al, Ga, or In) in the *P*6_4_22 phase via the method of GGA-PBE.

Planes	Materials	*E*_max_	*E*_min_	Ratio	Planes	Materials	*E*_max_	*E*_min_	Ratio
(001)	*P*6_4_22-AlP	120.333	120.333	1.000	(110)	*P*6_4_22-AlP	145.147	120.334	1.206
	*P*6_4_22-GaP	132.093	132.093	1.000		*P*6_4_22-GaP	151.508	119.008	1.273
	*P*6_4_22-InP	84.764	84.764	1.000		*P*6_4_22-InP	104.849	83.797	1.251
(011)	*P*6_4_22-AlP	142.751	120.334	1.186	(111)	*P*6_4_22-AlP	145.081	120.334	1.205
	*P*6_4_22-GaP	151.508	132.093	1.147		*P*6_4_22-GaP	151.508	132.093	1.147
	*P*6_4_22-InP	104.849	84.764	1.237		*P*6_4_22-InP	104.849	84.764	1.237
(100)	*P*6_4_22-AlP	145.147	120.334	1.206	(010)	*P*6_4_22-AlP	145.147	120.334	1.206
	*P*6_4_22-GaP	151.508	119.008	1.273		*P*6_4_22-GaP	151.508	119.008	1.273
	*P*6_4_22-InP	104.849	83.797	1.251		*P*6_4_22-InP	104.849	83.797	1.251

**Table 4:** Average mass per atom, *M*_a_/g; the transverse and longitudinal sound velocities, ν_t,_ ν_l_/(km∙s^−1^); the density of number of atom per volume, *p*; and the minimum thermal conductivity at high temperature, *κ*_min_/(W∙m^−1^∙K^−1^), of *P*6_4_22- and *F*$\bar{4}3$*m*-*X*P (*X* = Al, Ga, or In) base on calculated (GGA-PBE) Young’s modulus *E*, density of the crystal *ρ*, bulk moduli *B*, and shear moduli *G*.

	Clark		Cahill			
	*M_a_* × 10^−23^	*k*_min_	ν_t_	ν_l_	*p* × 10^28^	*k*_min_
P6_4_22-AlP	4.817	1.222	4.523	7.825	5.379	1.338
*F*$\bar{4}3$*m*-AlP	4.817	1.132	4.520	7.874	4.777	1.240
P6_4_22-GaP	8.389	0.972	3.381	5.569	6.047	1.058
*F*$\bar{4}3$*m*-GaP	8.389	0.904	3.836	6.291	4.779	1.024
P6_4_22-InP	12.126	0.610	2.885	5.115	3.666	0.669
*F*$\bar{4}3$*m*-InP	12.126	0.592	2.775	4.822	3.748	0.647
